# Sports participation and muscle mass affect sex-related differences in bone mineral density between male and female adolescents: A longitudinal study

**DOI:** 10.1590/1516-3180.2018.031040119

**Published:** 2019-05-08

**Authors:** Rafael Luiz-de-Marco, Han Kemper, Ricardo Ribeiro Agostinete, André Oliveira Werneck, Santiago Maillane-Vanegas, Yuri da Silva Faustino-da-Silva, Isabella Exupério, Rômulo Araújo Fernandes

**Affiliations:** I Master’s Student and Researcher of Physical Education, Laboratory of InVestigation in Exercise (LIVE), Department of Physical Education, Universidade Estadual Paulista (UNESP), and Postgraduate Student, Postgraduate Program on Physical Therapy, Department of Physical Therapy, UNESP, Presidente Prudente (SP), Brazil.; II PhD. Emeritus Professor, Amsterdam UMC, Amsterdam Public Health Research Institute, Amsterdam, Netherlands.; III MSc. Researcher, Laboratory of InVestigation in Exercise (LIVE), Department of Physical Education, Universidade Estadual Paulista (UNESP), Presidente Prudente (SP), Brazil.; IV Master’s Student and Researcher, Laboratory of InVestigation in Exercise (LIVE), Department of Physical Education, Universidade Estadual Paulista (UNESP), Presidente Prudente (SP), Brazil.; V MSc. Researcher,Laboratory of InVestigation in Exercise (LIVE), Department of Physical Education, Universidade Estadual Paulista (UNESP), and Doctoral Student, Postgraduate Program on Physical Therapy, Department of Physical Therapy, UNESP, Presidente Prudente (SP), Brazil.; VI Master’s Student and Researcher of Physical Education, Laboratory of InVestigation in Exercise (LIVE), Department of Physical Education, Universidade Estadual Paulista (UNESP), and Postgraduate Student, Postgraduate Program on Physical Therapy, Department of Physical Therapy, UNESP, Presidente Prudente (SP), Brazil.; VII Master’s Student and Researcher of Physical Education, Laboratory of InVestigation in Exercise (LIVE), Department of Physical Education, Universidade Estadual Paulista (UNESP), and Postgraduate Student, Postgraduate Program on Physical Therapy, Department of Physical Therapy, UNESP, Presidente Prudente (SP), Brazil.; VIII PhD. Associate Professor, Laboratory of InVestigation in Exercise (LIVE), Department of Physical Education, Universidade Estadual Paulista (UNESP); Associate Professor, Postgraduate Program on Physical Therapy, Department of Physical Therapy, UNESP; and Associate Professor, Postgraduate Program on Movement Sciences, Department of Physical Education, UNESP, Presidente Prudente (SP), Brazil.

**Keywords:** Adolescent, Bone and Bones, Growth and Development, Sports, Puberty

## Abstract

**BACKGROUND:**

Sports participation plays an important role in bone gain during childhood and adolescence. The aim here was to identify sex-related determinants of bone mineral density (BMD) differences between male and female adolescents, with emphasis on the role of sports participation.

**DESIGN AND SETTING:**

Longitudinal study conducted in a public university in Presidente Prudente, Brazil.

**METHODS:**

The sample comprised 48 adolescents aged 11-17 years, of both sexes, who were matched according to sex, age and sports participation. BMD was the main outcome, while muscle mass, sports participation, calendar age and biological maturation were treated as covariates. Participants were followed up after nine months.

**RESULTS:**

At baseline, BMD values were similar between the sexes. However, adjustment for covariates showed that BMD was higher among girls at all sites, with a contribution from lean soft tissue (LST) in the model (partial eta-squared, ES-r = 0.619 in upper limbs; 0.643 in lower limbs; 0.699 in spine; and 0.599 in whole body). Sports participation only explained the upper-limb variance (ES-r = 0.99). At the follow-up, the results resembled the baseline except in the lower limbs (P = 0.109), in which BMD was similar between the groups. BMD gain over time was similar between girls and boys in all segments, and baseline LST affected upper-limb and whole-body BMD accrual (ES-r = 0.396 and 0.107, respectively).

**CONCLUSION:**

Whole-body and specific-site BMD differed between baseline and follow-up. However,BMD accrual was similar between the sexes, given that muscle mass constituted the most relevant determinant of the difference between them.

## INTRODUCTION

Bone health has become a concern in modern society due to the economic burden and impairment in quality of life caused by osteoporosis.^1^ Osteoporotic fractures increase both healthcare costs and the risk of early mortality.^2^^,^^3^^,^^4^ Taking into account the epidemiology of osteoporosis, women exhibit greater risk of developing osteoporosis than do men. This greater risk for the female sex is strongly determined by specific events that occur during adolescence.^5^

The current literature shows how childhood and adolescence are complex phases that are critical periods for the development of bone mineral density (BMD) accrual. There is increasing acceptance of the hypothesis that osteoporosis may be a pediatric metabolic disease, with manifestations during adulthood.^6^ Therefore, the peak bone mass reached during this period is a determinant of BMD observed during adulthood, and this constitutes a relevant determinant of the risk of osteoporosis in adulthood.^7^

Among the variables capable of affecting bone health, sports participation plays an important role in the process of boosting bone gains during childhood and adolescence.^8^ Thus, it is important to consider sports and resistance training as relevant tools for improving bone mineral density (BMD)^8^^-^^9^ and joint stability, with consequently stronger bones.^11^ Another relevant fact is that the rate of sports participation may differ between boys and girls, such that it is higher among males.^9^^,^^10^^,^^11^^,^^12^^,^^13^ Moreover, during adolescence, there are several correlates affecting sex-related differences in gains of bone mineral content among athletes and non-athletes, such as maturation, muscle mass and dietary factors.^14^^,^^15^

The abovementioned background identifies a significant difference regarding bone density between boys and girls.^14^^,^^15^ However,it does not quantify the burden of each covariate in this process.

Therefore, the purpose of this nine-month longitudinal study was to identify determinants of sex-related BMD differences between male and female adolescents, and to identify the role of sports participation in this phenomenon. We hypothesized that bone density differences between boys and girls would disappear after controlling for these variables, and that sports participation would maintain its impact on bone density even after controlling for these confounders.

## METHODS

### Ethical considerations

The Research Ethics Board of the School of Science and Technology of São Paulo State University (Universidade Estadual Paulista, UNESP), in Presidente Prudente, Brazil, approved this study (procedural number 02891112.6.0000.5402, May 7, 2012). All parents/guardians of potential participants signed a written consent form before their children participated inthe study.

### Design, setting and participants

This longitudinal study (ABCD-Growth Study) was conducted over a nine-month period with two evaluations: at the baseline and at a follow-up after nine months. This nine-month period was determined through examining previous studies reported in the literature and through work done by our research group, in which it was identified that nine months of exercise practice/sports participation was sufficient for this to promote positive effects on bone health among adolescents.^9^^,^^16^^,^^17^

The study was conducted in the metropolitan region of the city of Presidente Prudente, which is in the western part of the state of São Paulo, with a population of 200,000 inhabitants and a human development index of 0.806.

The sample consisted of 48 adolescents aged between 9 and 17 years, who were matched according to sex (1:1 boy/girl pairs), chronological age and sports participation: nine pairs in a control group (n = 18) and 15 pairs in an impact sports practice group (n = 30). The adolescents in the sports participation group were engaged in impact sports (soccer and combat sports, i.e. karate and judo) and the control group were not engaged in any sports. In relation to the three sports, the adolescents in the impact sports group were matched according to their sport. The adolescents engaged in sports participation were contacted at private and public sports clubs located in the metropolitan area of the city. Thecontrol group was contacted in public and private schools in the metropolitan area of the city.

In the sports participation group, the inclusion criteria adopted were as follows: (i) engagement in only one sport over the previous 12 months; and (ii) the participant’s legal guardian had signed a written consent form. In the control group, the inclusion criteria adopted were: (i) six months without any engagement in organized sport outside school; (ii) participation in school physical education classes; (iii) no orthopedic disease that prevented the adolescent from practicing sports; (iv) not being pregnant; (v) the subject’s legal guardian had signed a written consent form.

### Dependent variable: bone mineral density

Whole body BMD (g/cm^2^) was estimated by means of dual-energy X-ray absorptiometry (DEXA) (GE model, Lunar-DPX-NT, United Kingdom). BMD measurements were made at the baseline and after nine months of study, in different body segments: upper limbs, lower limbs and spine; these were performed by a technician with extensive experience in these measurements. All the examinations were carried out in the same place at the university (UNESP). During the DEXA scans, the participants wore light clothes, were barefoot (with no metal on the body) and remained immobile for approximately 15 minutes. The results were generated by means of specific software that was provided with the equipment. The DEXA measurements at the baseline were repeated after nine months of follow-up.

### Covariates

Sex (male or female) and the quantity of lean soft tissue (LST) (measured using DEXA and expressed in kg) were treated as covariates. LST, composed of muscle mass and residuals, is a widely used surrogate measurement of muscle mass. Moreover,somatic maturation, through estimated peak height velocity (PHV) was used as an indicator of biological maturation. PHVwas estimated at baseline using anthropometric measurements (height),^18^ based on the mathematical models described by Moore et al.^19^ PHV identifies the time (in years) until (negative scores) or after (positive scores) the moment of greatest height accrual during adolescence. Sports participation was also considered to be a covariate. In the statistical models, sports participation was treated as follows: 0 = control and 2 = soccer/combat (high impact) sports.

### Statistical analysis

Mean and standard deviation (SD) values were used to describe the characteristics of the sample. Initially, crude comparisons between boys and girls were performed using Student’s t test for independent samples (top of the tables). Secondly, the same sex comparisons were simultaneously adjusted for all covariates (PHV, LST, sports participation and vitamin D score) using analysis of covariance (ANCOVA) at the baseline and at the follow-up. The models at the follow-up are presented as comparisons of BMD after nine months and comparisons of BMD percentage accruals after nine months. In the ANCOVA models, the descriptive statistics were composed of estimated marginal means and 95% confidence intervals (95% CI), and effect-size measurements were expressed as partial eta-squared measurements (ES-r). All statistical procedures were performed in the BioEstat software (version 5.0) and the significance level (P-value) was previously set at P < 0.05.

## RESULTS

The descriptive statistics are presented in Table 1. Theboys and girls presented similar body mass (P = 0.331) and height (P=0.118). However, the boys presented higher LST at the baseline (P-value = 0.001) and gained more LST after nine months (boys, 3.9 kg, versus girls, 2.2 kg; P = 0.001). Somaticmaturation (PHV) was different between the groups at the baseline (P=0.007) and the age at PHV was 13.2 years for boys and 12.0years for girls (P ≤ 0.001). In addition, the adolescent sports participants presented greater quantities of LST than did the control group (adjusted for sex and somatic maturation).

**Table 1.** Summary of the characteristics of the boys and girls at the baseline (n = 48)

	Boys (n = 24)	Girls (n = 24)	P-value
	Mean (SD)	Mean (SD)	
Numerical data			
Age (years)	12.0 (1.6)	12.1 (1.6)	1.000
Body mass (kg)	45.7 (12.0)	44.6 (14.7)	0.331
Height (cm)	154.7 (12.1)	149.5 (10.2)	0.118
PHV (years)	-1.2 (1.3)	0.9 (1.4)	0.007
APHV (years)	13.2 (0.4)	12.0 (0.5)	< 0.001
LST (kg)	34.7 (8.3)	28.6 (5.9)	0.001
LST gains (kg)	3.9 (2.2)	2.2 (1.3)	0.001
Categorical data	n (%)	n (%)	
Sports participation, n*			
Control, n	9 (50)	9 (50)	
Impact sports, n	15 (50)	15 (50)	

*Sports participation values refer to sample size per group. SD = standard deviation; PHV = peak height velocity; APHV = age peak height velocity; LST = lean soft tissue.

Thecomparisons of bone mineral density (BMD) at the baseline between the boys and girls are presented in Table 2. The crude analysis showed that the BMD values were similar between the boys and girls. In the multivariate models, after adjustment for covariates, the girls showed higher bone density in the upper limbs. This variance was explained in terms of sex (ES-r = 0.144, i.e. a high effect size), LST (ES-r = 0.619, i.e. a high effect size) and sports participation (ES-r = 0.099, i.e. a moderate effect size). The spinal variance was explained in terms of sex (ES-r = 0.198, i.e. a high effect size) and LST (ES-r = 0.643, i.e. a high effect size). For the lower limbs and whole body, the variance was explained in terms of sex (ES-r = 0.081, i.e. a moderate effect size for the lower limbs, and ES-r = 0.207, i.e. a high effect size for the whole body) and LST (ES-r = 0.699, i.e. a high effect size for the lower limbs, and ES-r = 0.599, i.e. a high effect size for the whole body).

**Table 2.** Comparisons of bone mineral density (BMD) at the baseline, between the boys and girls (n = 48)

BMD (g/cm^2^)	Boys (n = 24)	Girls (n = 24)	Sex	PHV	LST	Sport
	Mean (SD)	Mean (SD)	P-value			
Upper limbs	0.707 (0.079)	0.707 (0.089)	0.992	---	---	---
Spine	0.907 (0.121)	0.930 (0.171)	0.599	---	---	---
Lower limbs	1.112 (0.136)	1.062 (0.118)	0.184	---	---	---
Whole body	1.014 (0.088)	1.013 (0.103)	0.963	---	---	---
BMD (g/cm^2^)	Mean (95% CI)	Mean (95% CI)	Effect size determined through ANCOVA (eta-squared [ES-r])			
Upper limbs	0.676 (0.648 to 0.704)	0.740 (0.712 to 0.786)	0.144*	0.008	0.619*	0.099*
Spine	0.851 (0.802 to 0.901)	0.986 (0.937 to 1.035)	0.198*	0.005	0.643*	0.030
Lower limbs	1.056 (1.017 to 1.095)	1.119 (1.080 to 1.158)	0.081*	0.025	0.699*	0.024
Whole body	0.966 (0.932 to 1.000)	1.062 (1.028 to 1.096)	0.207*	0.063	0.599*	0.003

*Denotes covariate with P-value < 0.05. SD = standard deviation; 95% CI = 95% confidence interval; PHV = peak height velocity; LST = lean soft tissue.

At the nine-month follow-up, the boys did not present higher BMD values in the crude analysis (Table 3). In the multivariate equation, while sex better explained the model for the upper limbs (ES-r = 0.171, i.e. a high effect size), spine (ES-r = 0.275, i.e. a high effect size) and whole body (ES-r = 0.241, i.e. a high effect size), LST significantly explained the variance observed in the upper limbs (ES-r = 0.807, i.e. a high effect size), spine (ES-r = 0.681, i.e. a high effect size), lower limbs (ES-r = 0.616, i.e. a high effect size) and whole body (ES-r = 0.681, i.e. a high effect size), which were significantly higher among the girls.

**Table 3.** Comparisons of bone mineral density (BMD) after nine months of follow-up, between the boys and girls (n = 48)

BMD (g/cm^2^)	Boys (n = 24)	Girls (n = 24)	Sex	PHV	LST	Sport
	Mean (SD)	Mean (SD)	P-value			
Upper limbs	0.766 (0.122)	0.747 (0.095)	0.175	---	---	---
Spine	0.968 (0.145)	1.001 (0.176)	0.238	---	---	---
Lower limbs	1.163 (0.139)	1.110 (0.118)	0.481	---	---	---
Whole body	1.058 (0.100)	1.053 (0.105)	0.535	---	---	---
BMD (g/cm^2^)	Mean (95% CI)	Mean (95% CI)	Effect size determined through ANCOVA (eta-squared [ES-r])			
Upper limbs	0.724 (0.967 to 0.750)	0.790 (0.764 to 0.817)	0.171*	0.001	0.807*	0.045
Spine	0.900 (0.850 to 0.950)	1.070 (1.020 to 1.121)	0.275*	0.019	0.681*	0.053
Lower limbs	1.106 (1.062 to 1.151)	1.168 (1.123 to 1.212)	0.059	0.028	0.616*	0.016
Whole body	1.006 (0.974 to 1.038)	1.106 (1.074 to 1.139)	0.241*	0.070	0.681*	0.005

*Denotes covariate with P-value < 0.05. SD = standard deviation; 95% CI = 95% confidence interval; PHV = peak height velocity; LST = lean soft tissue.

Comparison of BMD accrual over the nine-month period between the boys and girls (Table 4) showed that the gains were similar between the boys and girls and that BMD accrual was not significantly affected by sex. On the other hand, baseline LST was the variable that most showed statistically significant differences, in the upper limbs (ES-r = 0.396, i.e. a high effect size) and whole body (ES-r = 0.107, i.e. a moderate effect size). 

**Table 4.** Comparisons of bone mineral density (BMD) percentage accruals after nine months of follow-up, between the boys and girls (n = 48)

BMD (g/cm^2^)	Boys (n = 24)	Girls (n = 24)	Sex	PHV	LST	Sport
	Mean (SD)	Mean (SD)	P-value			
Upper limbs	7.9 (7.7)	5.6 (4.1)	0.062	---	---	---
Spine	6.6 (5.0)	7.8 (4.2)	0.741	---	---	---
Lower limbs	4.7 (3.7)	4.5 (2.6)	0.186	---	---	---
Whole body	4.2 (2.2)	3.9 (2.2)	0.819	---	---	---
BMD (g/cm^2^)	Mean (95% CI)	Mean (95% CI)	Effect size determined through ANCOVA (eta-squared [ES-r])			
Upper limbs	7.0 (4.3 to 9.7)	6.5 (3.8 to 9.3)	0.001	0.018	0.396*	0.036
Spine	5.8 (3.2 to 8.4)	8.6 (6.0 to 11.2)	0.037	0.010	0.018	0.012
Lower limbs	4.8 (3.0 to 6.6)	4.4 (2.6 to 6.1)	0.002	0.003	0.065	0.004
Whole body	4.0 (2.8 to 5.2)	4.2 (2.9 to 5.4)	0.000	0.001	0.107*	0.001

*Denotes covariate with P-value < 0.05. SD = standard deviation; 95% CI = 95% confidence interval; PHV = peak height velocity; LST = lean soft tissue.

## DISCUSSION

The aim of the present study was to identify determinants of sex-related BMD differences between male and female adolescents, and to identify the role of sports participation in this phenomenon. The findings suggest that the quantity of lean soft tissue is the most relevant determinant of bone-related differences between boys and girls, while sports participation indirectly affects bone density, in the sense that the more sports practice that was performed, the greater the gain in lean soft tissue would be.

In the present study, the most relevant variable relating to sex-specific differences in bone density was lean soft tissue (both baseline values ​​and gains over nine months). Previous studies have identified a positive relationship between muscle mass and bone variables among adolescents.^20^^,^^21^ The strengthening and bone remodeling that are stimulated by muscle contraction seems to be explained by the daily tension that muscles exert on the bone structure.^12^^,^^22^^,^^23^ In agreement with our findings, the literature shows evidence that muscle development is an important factor for bone adaptation.^16^ Expanded muscle mass contributes towards greater muscle strength and, subsequently, increased mechanical stress in the bone, thus stimulating bone adaptation.^24^ Although bone undergoes constant adaptation through the processes of modeling and remodeling, mechanical tension stimulates physiological mechanisms that can influence bone formation. Therefore,mechanical stresses can trigger a cascade of events through mechanotransduction.^20^

Regular participation in weight-bearing sports during adolescence is linked to bone density gains.^9^ Stronger bones reduce the likelihood of pathological fractures.^11^ Although our findings did not reveal that sport had any direct role in the bone mineral density results, sports participation is beneficial for gain and maintenance of muscle mass.^25^ Muscle mass has been shown to be the main pathway through which physical exercise/sports participation can improve part of the osteogenic process.^26^^,^^27^ However, there are other pathways, such as physical loading and the ground reaction force generated by the activity.

In fact, regarding sex-related bone differences, sports participation and muscle mass acted independently in the upper limbs, spine and whole body, and the stress component may explain this. Recently, Ito et al.^16^ found that judo participation increased BMD accrual in the spine more than was seen among their control group, but only among boys. This is interesting because, in our study, after excluding the impact of muscle mass and sports participation, the girls presented higher values in the spine than the boys (both at the baseline and at the follow-up), thus denoting the significant participation of both muscle mass and sports participation in this phenomenon. Moreover, judo, karate and swimming are sports in which the upper limbs are highly required,^16^ which helps to explain the significant impact of these sports on this body segment. Similarly, Agostinete et al.^16^ suggested that the potentially harmful impact of swimming on bone formation in adolescents would be attenuated by its effect on the upper limbs, given the high amount of muscle force produced in this body segment.

In the pre-pubertal and pubertal periods, hormonal and maturational factors seem to promote greater bone accrual attributable to physical exercise among boys than among girls.^16^ The independent impact of sports participation on sex-related BMD differences appears to agree with the assumption that both the effects of exercise and sex-related effects offer greater bone growth in boys than in girls. Taking that into consideration, the low magnitude of theeffect attributable to sports participation may be related to the sports category divisions used in the present study. Therefore,to better understand this important issue, future studies should identify sex-related BMD differences in specific sports, instead of combining sports with different impacts into a single group.

In the present study, we found that somatic maturation did not have any impact on the models. It has been hypothesized that biological maturation affects bone mineral density in both sexes.^28^^,^^29^ However, given that the subjects in the present study had a mean chronological age of 12 years, the girls were mostly within the somatic maturation band, while the boys were still around one year away from the peak. Thus, the differences between the sexes could explain part of the variation in somatic maturation. Moreover, because we also adjusted for lean soft tissue, which is affected by biological maturation in both sexes,^30^^,^^31^ it is plausible that somatic maturation affects lean soft tissue and that lean soft tissue affects BMD. In this manner, somatic maturation might still act as a mediator, which would thus restore its effect.

Although the present study presented positive points such as its longitudinal design, matching of the sample according to sex and age and analysis of BMD using DEXA, some limitations ofthis study need to be recognized. The first relates to the absenceof measurement of bone geometry (a concept that characterizes both bone mineral density and bone mineral content), which would have significantly complemented the bone density measurements. Likewise, follow-ups longer than nine months would have been interesting, with the aim of observing greater potential differences between boys and girls, as well as for identifying manifestations of maturational events. The absence of analyses regarding hormones needs to be recognized, given the impact of hormones on bone formation in both genders.

Moreover, estimates using the somatic maturation indicator may present some bias, although previous studies have pointed out that the optimal chronological age for estimating PHV is between 10 years and 14 years for girls and between 12 years and 16 years for boys,^32^ which is concordant with what was done in our sample. In this regard, future studies should also use better methods for making predictions of biological maturation, such as skeletal age.^33^

Although the adolescents who were included in the control group were only attending school physical education classes and were not performing any type of sport, it needs to be borne in mind that there was a lack of information regarding these adolescents’ habitual physical activity. In addition, unfortunately, we did not perform any sample size calculations for this study, which was based on a convenience sample from a larger cohort study by our group.

Lastly, measurements of calcium consumption and exposure to sunlight (vitamin D) would be interesting confounders to insert in the multivariate models presented here.

## CONCLUSION

The present findings suggest that lean soft tissue is the most relevant determinant of the differences in BMD between boys and girls, while sports participation and somatic maturation potentially have indirect roles in sex-related differences in BMD over time. The main message from this study is that, through engagement in sports practice, female adolescents may present increases in lean soft tissue, which in turn positively influence their BMD gain, thus enabling BMD values similar to or greater than among males.
